# A Novel Highly Thermostable Multifunctional Beta-Glycosidase from Crenarchaeon *Acidilobus saccharovorans*

**DOI:** 10.1155/2015/978632

**Published:** 2015-10-11

**Authors:** Vadim M. Gumerov, Andrey L. Rakitin, Andrey V. Mardanov, Nikolai V. Ravin

**Affiliations:** Centre “Bioengineering”, Russian Academy of Sciences, Moscow 117312, Russia

## Abstract

We expressed a putative *β*-galactosidase Asac_1390 from hyperthermophilic crenarchaeon *Acidilobus saccharovorans* in *Escherichia coli* and purified the recombinant enzyme. Asac_1390 is composed of 490 amino acid residues and showed high sequence similarity to family 1 glycoside hydrolases from various thermophilic Crenarchaeota. The maximum activity was observed at pH 6.0 and 93°C. The half-life of the enzyme at 90°C was about 7 hours. Asac_1390 displayed high tolerance to glucose and exhibits hydrolytic activity towards cellobiose and various aryl glucosides. The hydrolytic activity with *p*-nitrophenyl (pNP) substrates followed the order pNP-*β*-D-galactopyranoside (328 U mg^−1^), pNP-*β*-D-glucopyranoside (246 U mg^−1^), pNP-*β*-D-xylopyranoside (72 U mg^−1^), and pNP-*β*-D-mannopyranoside (28 U mg^−1^). Thus the enzyme was actually a multifunctional *β*-glycosidase. Therefore, the utilization of Asac_1390 may contribute to facilitating the efficient degradation of lignocellulosic biomass and help enhance bioconversion processes.

## 1. Introduction

Lignocellulosic biomass, predominantly composed of cellulose, hemicellulose, and lignin, is the most abundant renewable resource on earth, and its degradation to soluble sugars is a key issue for the production of biobased chemicals and biofuels. Cellulose and hemicellulose consist of polysaccharides, and because of their recalcitrance, the enzymatic conversion of these substrates into simple sugars requires many steps.

Microorganisms capable of degrading cellulose possess three types of enzymes which act synergistically [1, 2]: (1) endoglucanases (EC 3.2.1.4), (2) exoglucanases also known as cellobiohydrolases (EC 3.2.1.91), and (3) *β*-glucosidases (EC 3.2.1.21). Endoglucanases randomly cleave the internal bonds of cellulosic materials, releasing oligosaccharides of various lengths and thus generating new ends of the polysaccharide chains. The cellobiohydrolases act progressively on these chain ends generating short cellooligosaccharides or cellobiose. *β*-Glucosidases hydrolyze cellooligosaccharides or cellobiose to glucose units [3, 4]. Hemicellulose is broken down into soluble xylose or other types of monosaccharides by hemicellulases such as endo-1,4-*β*-xylanase (EC 3.2.1.8), *β*-xylosidase (EC 3.2.1.37), *α*-L-arabinofuranosidase (EC 3.2.1.55), endo-1,4-*β*-mannanase (EC 3.2.1.78), *β*-mannosidase (EC 3.2.1.25), and *β*-galactosidase (EC 3.2.1.23) [5].

The presence of *β*-glucosidases is very important in cellulose hydrolysis process because they perform a rate-limiting step by preventing the accumulation of cellobiose, which inhibits the activities of most endo- and exoglucanases [6]. Moreover, most *β*-glucosidases are inhibited by glucose [7]. Therefore, *β*-glucosidases, with high specific activity and glucose tolerance, could improve the efficiency of cellulolytic enzyme complexes.

Thermostable enzymes have several advantages in lignocellulose degradation processes because they are able to withstand rough reaction conditions at elevated temperature and allow elongated hydrolysis time due to higher stability and reduced risk of contamination. High temperature promotes high activity of these enzymes and increases the solubility of substrates in the aqueous phase. Although a number of thermostable *β*-glucosidases have been identified [8–16], there are only several examples of glucose-tolerant thermostable *β*-glucosidases [13, 14].

*Acidilobus saccharovorans *345-15^T^ (DSM 16705) is an anaerobic, organotrophic, thermoacidophilic crenarchaeon with a pH range from 2.5 to 5.8 (optimum at pH 3.5 to 4) and a temperature range from 60 to 90°C (optimum at 80 to 85°C), isolated from an acidic hot spring of Uzon Caldera, Kamchatka, Russia [17]. The complete genome of* A. saccharovorans *was sequenced [18], and the presence of genes for extra- and intracellular glycoside hydrolases correlates with the growth of* A. saccharovorans *on carbohydrates. Particularly, the gene Asac_1390 encoding putative *β*-galactosidase (EC 3.2.1.23) was identified [18]. In this paper we report the cloning, expression, and biochemical characterization of this enzyme appearing to be a multifunctional *β*-glycosidase.

## 2. Materials and Methods

### 2.1. Cloning of* A. saccharovorans* Gene Asac_1390

The *β*-galactosidase gene Asac_1390 was amplified from* A. saccharovorans* genomic DNA by PCR using primers F2_NcoI (TTCCATGGCAGTTACCTTCCCAAA) and R2_BglII (5′-TTAGATCTGGATCTACCAGGCGCT-3′); the PCR products were digested with NcoI and BglII and inserted into pQE60 (Qiagen) at NcoI and BglII sites, yielding the plasmid pQE60_Asac1390.

### 2.2. Expression and Purification of Recombinant Enzyme Asac_1390

Plasmid pQE60_Asac1390 was transformed into* Escherichia coli* strain DLT1270 carrying plasmid pRARE2 (Novagen). Recombinant strain was grown at 37°C in Luria-Bertani medium (LB) supplemented with ampicillin and induced to express recombinant xylanases by adding isopropyl-*β*-D-thiogalactopyranoside (IPTG) to a final concentration of 1.0 mM at OD_600_ approximately 0.6 and incubated further at 37°C for 20 hours. The grown cells were harvested by centrifugation at 5,000 g for 20 min at 4°C, washed with 0.1 M sodium phosphate buffer, pH 7.0, and resuspended in 20 mL of the same buffer with lysozyme (1 mg/mL). The cells were incubated 30 min at 4°C and then disrupted by sonication. The insoluble debris was removed by centrifugation at 5,000 g for 20 min at 4°C. The supernatant was then incubated in a water bath for 30 min at 75°C and then for 30 min at 85°C (double heat treatment) and cooled on ice. Denatured proteins of* E. coli* were removed by centrifugation at 12,000 g for 20 min at 4°C. The protein sample was dialysed against 25 mM phosphate buffer (pH 7.0) at 4°C for 3 h.

The purity of the purified protein was examined by SDS-PAGE (10%) and its concentration was determined by the Bradford method using bovine serum albumin (BSA) as a standard.

### 2.3. Assay of *β*-Galactosidase Activity

The *β*-galactosidase activity was analyzed using* o*-nitrophenyl-*β*-D-galactopyranoside (oNPGal) as substrate following modified Craven method [19]. 1.076 mL of the substrate solution (0.7 mg/mL oNPGal in 0.1 M sodium phosphate buffer, pH 7.0) was preincubated at the appropriate temperature for 5 min, and the reaction was initiated by the addition of 0.05 mL of enzyme (0.08 *μ*g). The reaction was stopped with 0.375 mL of cold 1 M Na_2_CO_3_ solution. A blank, containing 0.05 mL of 0.1 M sodium phosphate buffer instead of enzyme solution, was used to correct for the thermal hydrolysis of oNPGal. The amount of released* o*-nitrophenol was measured at 420 nm. One unit (U) of enzyme activity was defined as the amount of enzyme required to liberate 1 *μ*mol of* o*-nitrophenol per min under described conditions.

### 2.4. Biochemical Characterization of Recombinant Enzyme Asac_1390

To examine the effects of pH and temperature on *β*-galactosidase activity, pH values were varied from 3.0 to 8.0 at 80°C using 0.1 M acetate buffer (pH 3.0 to 5.0) and 0.1 M sodium phosphate buffer (pH 6.0 to 8.0). Similarly, *β*-galactosidase assay was done at various temperatures (50–100°C) and pH 7.0 to determine the optimum temperature for Asac_1390 activity.

The thermostability of Asac_1390 was assessed by preincubating the enzyme at 90°C in sodium phosphate buffer, pH 7.0. The samples were collected at the desired intervals and assayed for the residual *β*-galactosidase activity in 0.1 M sodium phosphate buffer (pH 7.0) at 50°C.

The substrate specificity of Asac_1390 was determined using oNPGal,* p*-nitrophenyl-*β*-D-galactopyranoside (pNPGal),* p*-nitrophenyl-*β*-D-glucopyranoside (pNPGlu),* p*-nitrophenyl-*β*-D-xylopyranoside (pNPXyl), and pNP-*β*-D-mannopyranoside (pNPMan). The reactions were performed with 2.21 mM of each substrate in 0.1 M sodium phosphate buffer (pH 7.0) at 93°C and the activity was measured by release of pNP (at 405 nm) and oNP (at 420 nm).

When cellobiose was used as a substrate (0.5% w/v), the amount of glucose released was determined with a Sucrose/D-Glucose/D-Fructose Kit (R-Biopharm AG, Germany) according to the manufacturer's protocol. The cellobiose hydrolysis reactions were performed in 0.1 M sodium phosphate buffer (pH 6.0) at 50°C. In this assay, 1 U of activity is defined as the amount of enzyme which is required to release 1 *μ*mol of glucose per minute under test conditions.

Various concentrations of oNPGal, pNPGal, pNPGlu, pNPXyl, and pNPMan (from 0.277 to 2.21 mM for oNPGal, pNPGal, pNPGlu, and pNPMan and from 0.074 to 1.23 mM for pNPXyl) were used to determine kinetic parameters of Asac_1390. The reactions were performed in 0.1 M sodium phosphate buffer (pH 7.0) at 93°C. The enzyme kinetic parameters, *K*_*m*_ (mM), *V*_max_ (U/mg), *k*_cat_ (c^−1^), and *k*_cat_/*K*_*m*_, were calculated from Hanes-Woolf plot of Michaelis-Menten equation.

The effects of glucose on *β*-galactosidase activity were investigated at the concentrations of glucose from 10 to 100 mM using 2.21 mM oNPGal at 80°C in 0.1 M phosphate buffer (pH 7.0). The relative activity was defined as the relative value to the maximum activity without glucose. The type of inhibition and inhibition constants for glucose were determined by fitting to Cornish-Bowden and Dixon plots [20] using various concentrations of glucose (from 0 to 400 mM) with various concentrations of oNPGal (from 0.05 to 0.5 mM) as a substrate.

## 3. Results and Discussion

### 3.1. Gene Cloning and Enzyme Expression

The amino acid sequence of a putative *β*-galactosidase Asac_1390 from* A. saccharovorans* exhibited 54–71% identities with the glycoside hydrolases from the thermophilic archaea of the genera* Caldivirga*,* Sulfolobus*,* Vulcanisaeta*,* Thermoproteus*,* Ignisphaera*,* Thermoplasma*,* Thermosphaera*,* Picrophilus*,* Thermococcus*, and* Pyrococcus*, annotated as *β*-galactosidases or *β*-glucosidases. A Blastp search of the amino acid sequence of Asac_1390 suggested that residues 1–46 contain a signature typical of glycosyl hydrolase (GH) family 1 (Pfam00232). Particularly, Asac_1390 is 63% identical to *β*-glycosidase from* Sulfolobus acidocaldarius* that was found to exhibit activities toward *β*-glucosides, *β*-galactosides, and *β*-fucosides [15]. The results suggest that the enzyme could have broad substrate specificity. Asac_1390 does not have a predicted secretion signal suggesting an intracellular action of this enzyme.

**Figure 1:**
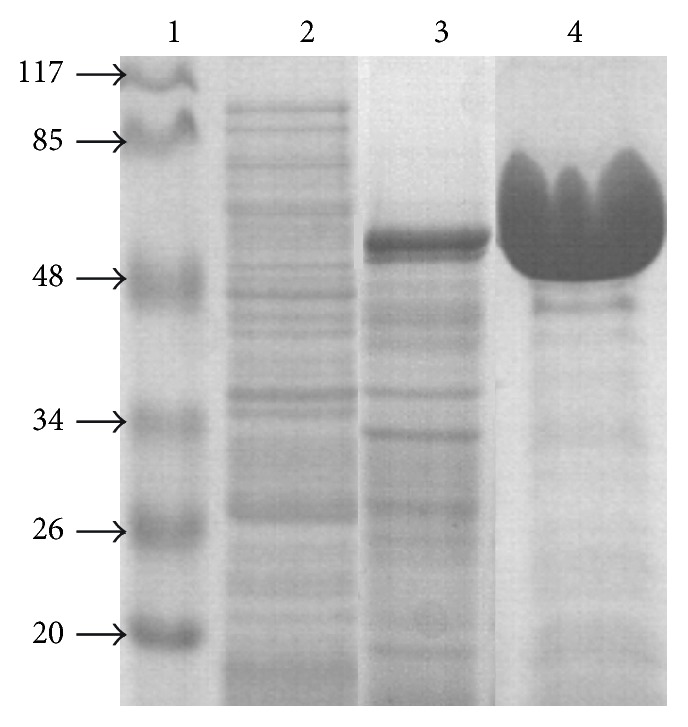
Expression and purification of recombinant glycosidase Asac_1390. SDS-PAGE was done using a 10.0% polyacrylamide gel; proteins were stained with Coomassie Brilliant Blue R-250. Lanes: 1—molecular weight markers (sizes are shown in kDa); 2—total protein from uninduced cells; 3—total protein from induced cells; 4—purified recombinant Asac_1390.

Asac_1390 gene with the same sequence as that reported in GenBank (CP001742) was cloned and expressed in* E. coli*. The recombinant enzyme was expressed with a yield of about 30% of the total soluble protein and was purified from crude* E. coli* extracts via two-step heat treatment to a purity of above 95%. The purified protein appeared in SDS-PAGE analysis as a single band with a molecular mass of approximately 55 kDa (Figure 1), consistent with the calculated value of 55,521 kDa based on the 490 amino acid residues of Asac_1390.

### 3.2. Effects of pH and Temperature on the Enzyme Activity

**Figure 2:**
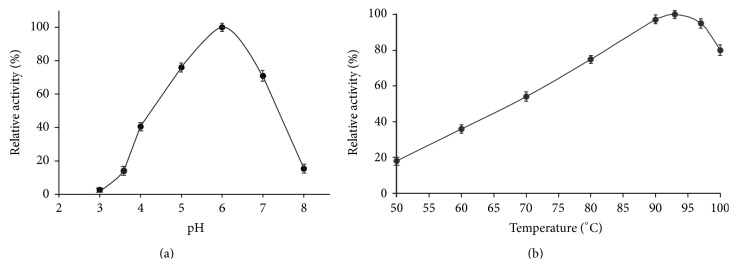
Effects of pH (a) and temperature (b) on the *β*-galactosidase activity of recombinant Asac_1390. Data represent the means of three experiments and error bars represent standard deviation.

The *β*-galactosidase activity was examined over a pH range of 3.0 to 8.0 at 80°C. Maximum activity was observed at pH 6.0 in sodium phosphate buffer. At pH 5.0 and 7.0, the activity was approximately 70% of the maximum (Figure 2(a)). The effect of temperature on enzyme activity was investigated in 0.1 M sodium phosphate buffer, pH 7.0 (Figure 2(b)). The maximum activity was recorded at 93°C; at temperature of 100°C the activity was about 80% of the maximum.

**Figure 3:**
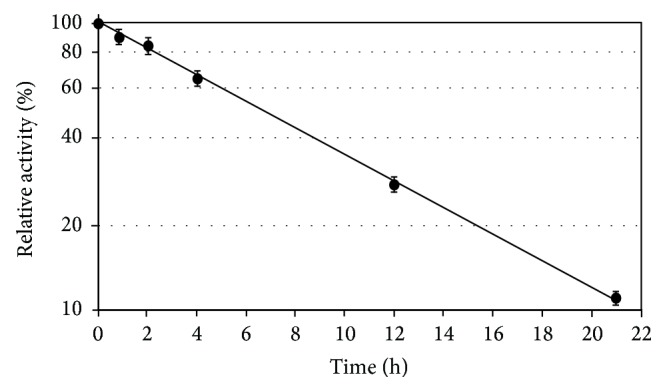
Thermal inactivation of recombinant Asac_1390. Purified Asac_1390 was preincubated at 90°C followed by *β*-galactosidase assay. Data represent the means of three experiments and error bars represent standard deviation.

The thermal stability of Asac_1390 was examined by measuring the activity over time at 90°C. The samples were withdrawn at various time intervals and assayed in 0.1 M sodium phosphate buffer (pH 7.0) at 50°C for 20 min (Figure 3). The Asac_1390 enzyme appeared to be extremely thermostable; the half-life of the enzyme at 90°C was about 7 hours.

These values of the temperature and pH optima of Asac_1390 are typical for *β*-galactosidases and *β*-glucosidases from hyperthermophilic archaea, including enzymes from* Sulfolobus solfataricus* (95°C and pH 6.5, [21]),* Pyrococcus furiosus* (100°C and pH 5.0, [22]),* S. acidocaldarius* (90°C and pH 5.5, [15]), and* Thermococcus kodakarensis* (100°C and pH 6.5, [23]). In terms of thermal inactivation, Asac_1390 is one of the most thermostable *β*-glycosidases with the half-life of 7 h at 90°C. It showed a higher stability than *β*-glycosidases from* S. solfataricus* and* S. acidocaldarius*, but a lower stability than those from* P. furiosus* (85 h at 100°C) and* T. kodakaraensis* (18 h at 90°C).

### 3.3. Effect of Glucose on the Activity of Asac_1390

The effects of glucose on *β*-galactosidase activity during oNPGal hydrolysis were investigated at various concentrations of D-glucose. The addition of 10, 50, and 100 mM of sugar reduced the activity of Asac_1390 to 80%, 76%, and 65%, respectively. Thus, glucose had little effect on hydrolytic activity.

The Cornish-Bowden and Dixon plots demonstrated a mixed type of inhibition of Asac_1390 by glucose. The dissociation constant of the EIS complex (*K*_*i*_′ = 158 mM) was significantly lower than that for EI complex (*K*_*i*_ = 500 mM). Glucose was reported to be a competitive inhibitor of *β*-glycosidase from* S. solfataricus* [21] with the inhibition constant *K*_*i*_ of 96 mM, while it has little effect on the *β*-glucosidase from* Pyrococcus furiosus* with an apparent *K*_*i*_ of 300 mM [22].

### 3.4. Substrate Specificity and Kinetics of Asac_1390

The hydrolytic activity of Asac_1390 was investigated with various aryl glycosides (Table 1). For the pNP substrates the highest activity was observed for pNPGal, followed by pNPGlu and pNPXyl. The hydrolysis of pNPMan was the least effective. The activity of the enzyme for oNPGal was about the same as for pNPGal, indicating that the enzyme equally and efficiently hydrolyzed *β*-1-2 and *β*-1-4 linkages. The Michaelis-Menten constants (*K*_*m*_), turnover numbers (*k*_cat_), and catalytic efficiencies (*k*_cat_/*K*_*m*_) for oNPGal, pNPGal, pNPGlu, pNPXyl, and pNPMan are presented in Table 2. The value of *k*_cat_/*K*_*m*_ for pNPGlu was much higher than that obtained with pNPGal, indicating that Asac_1390 was not a *β*-galactosidase as it was annotated, but rather a *β*-glucosidase.

The Asac_1390 enzyme has high specific *β*-galactosidase and *β*-glucosidase activity (246–328 U mg^−1^ on different substrates), a high affinity with substrate (*K*_*m*_ = 0.24 mM for pNPGlu), and high catalytic activity (*k*_cat_/*K*_*m*_ = 1327 s^−1^ mM^−1^ for pNPGlu). These values are among the highest among archaeal enzymes of this class. Some more active *β*-galactosidases from thermophilic bacteria are known, but usually they are less thermostable (e.g., 30,400 U mg^−1^ and half-life of 1.5 h at 90°C in case of *β*-glucosidase* Thermotoga petrophila* [24]).

Taking into account that some microbial GH1 family *β*-glycosidases had low specific activity for cellobiose, we investigated the hydrolytic activity of Asac_1390 with this natural *β*-glucosidase substrate. The observed activities of Asac_1390 with cellobiose and pNPGlu at 50°C and pH 6.0 were 68 U mg^−1^ and 53 U mg^−1^, respectively. Typically, glucose-tolerant microbial *β*-glucosidases have considerably lower specific activity for cellobiose than for pNPGlu, while the known exceptions (e.g., *β*-glucosidase from* Thermoanaerobacterium thermosaccharolyticum*) are less thermostable than Asac_1390 [14].

The broad substrate specificity of Asac_1390 is an interesting and important feature of this enzyme. Although initially annotated as *β*-galactosidase, this enzyme exhibits hydrolytic activity towards cellobiose and various aryl glucosides: high activity with pNPGal and pNPGlu, followed with pNPXyl and pNPMan. Such broad substrate specificity *β*-glycosidases are known among Archaea [25], including* S. acidocaldarius* and* S. solfataricus* [15, 26]. Analysis of recently determined three-dimensional structure of Asac_1390 [27] could help to reveal molecular features defining substrate specificity of the enzyme.

Multifunctionality of Asac_1390 makes it very promising for application in enzymatic hydrolysis of lignocellulose biomass. *β*-Xylosidases and *β*-glucosidases are responsible for the last steps of the hydrolysis of xylan and cellulose: cleavage of xylobiose to xylose [28] and cellobiose to glucose [1]. The activity of *β*-mannosidase is also useful since this enzyme participates in the production of fermentable sugar from another component of hemicellulosic materials, mannan [29]. For instance,* Trichoderma reesei* is a well-known cellulase-overproducing filamentous fungus which secretes several cellulolytic enzymes. However, *β*-glucosidase activity in* T. reesei* is partly mycelium-bound and obviously limits the enzyme performance in commercial* T. reesei *preparations [30]. Supplementation of enzyme complex produced by* T. reesei* with highly active *β*-glucosidase, exhibiting also *β*-xylosidase, *β*-mannosidase, and *β*-galactosidase hydrolytic activities, could improve efficiency of processing of lignocellulose biomass. Asac_1390 is particularly suitable for these purposes due to its resistance to inhibition by the main reaction product, glucose.

## 4. Conclusions

In this study, we expressed and characterized a thermostable recombinant glycosyl hydrolase Asac_1390 from* A. saccharovorans.* This enzyme is optimally active at high temperature (93°C) and pH 6.0 and is highly thermostable. Asac_1390 is a multifunctional *β*-glycosidase exhibiting activities of *β*-glucosidase, *β*-galactosidase, *β*-xylosidase, and *β*-mannosidase. The broad substrate specificity and resistance to inhibition by glucose make the new enzyme promising for application in enzymatic degradation of lignocellulosic materials.

## Figures and Tables

**Table 1:** Hydrolytic activity of Asac_1390 with different substrates.

Substrate	Specific activity (U mg^−1^)
oNPGal	325 ± 10
pNPGal	328 ± 10
pNPGlu	246 ± 6
pNPXyl	72 ± 1
pNPMan	28 ± 2

The reaction was carried out in 100 mM sodium phosphate buffer (pH 7.0) at 93°C. Data represent the means of three separate experiments.

**Table 2:** Kinetic parameters of Asac_1390.

Substrate	*K*_*m*_ (mM)	*k*_cat_ (s^−1^)	*k*_cat_/*K*_*m*_ (s^−1^ mM^−1^)	*V*_max⁡_ (U mg^−1^)
oNPGal	2.0	434.0	217.0	468.9
pNPGal	2.9	461.8	159.8	499.3
pNPGlu	0.24	318.5	1327.1	343.9
pNPXyl	4.65	114.6	24.6	123.5
pNPMan	2.4	58.2	24.2	62.8

The reaction was carried out in 100 mM sodium phosphate buffer (pH 7.0) at 93°C. Data represent the means of three separate experiments.

## References

[1] Béguin P. (1990). Molecular biology of cellulose degradation. *Annual Review of Microbiology*.

[2] Wood T. M., McCrae S. I., Bhat K. M. (1989). The mechanism of fungal cellulase action. Synergism between enzyme components of *Penicillium pinophilum* cellulase in solubilizing hydrogen bound-ordered cellulose. *The Biochemical Journal*.

[3] Ryu D. D. Y., Mandels M. (1980). Cellulases: biosynthesis and applications. *Enzyme and Microbial Technology*.

[4] Wood T. M. (1985). Properties of cellulolytic enzyme systems. *Biochemical Society Transactions*.

[5] Zhang Y.-H. P., Himmel M. E., Mielenz J. R. (2006). Outlook for cellulase improvement: screening and selection strategies. *Biotechnology Advances*.

[6] Xin Z., Yinbo Q., Peiji G. (1993). Acceleration of ethanol production from paper mill waste fiber by supplementation with *β*-glucosidase. *Enzyme and Microbial Technology*.

[7] Hong J., Ladisch M. R., Gong C., Wankat P. C., Tsao G. T. (1981). Combined product and substrate inhibition equation for cellobiase. *Biotechnology and Bioengineering*.

[8] Aït N., Creuzet N., Cattanéo J. (1979). Characterization and purification of thermostable *β*-glucosidase from *Clostridium thermocellum*. *Biochemical and Biophysical Research Communications*.

[9] Klippel B., Antranikian G., Horikoshi K. (2011). Lignocellulose converting enzymes from thermophiles. *Extremophiles Handbook*.

[10] Li X.-H., Bhaskar R., Yang H.-J., Wang D., Miao Y.-G. (2009). Screening and identification of new isolate: thermostable *Escherichia coli* with novel thermoalkalotolerant cellulases. *Current Microbiology*.

[11] Patchett M. L., Daniel R. M., Morgan H. W. (1987). Purification and properties of a stable *β*-glucosidase from an extremely thermophilic anaerobic bacterium. *Biochemical Journal*.

[12] Voorhorst W. G. B., Eggen R. I. L., Luesink E. J., de Vos W. M. (1995). Characterization of the celB gene coding for *β*-glucosidase from the hyperthermophilic archaeon *Pyrococcus furiosus* and its expression and site-directed mutation in *Escherichia coli*. *Journal of Bacteriology*.

[13] Jabbour D., Klippel B., Antranikian G. (2012). A novel thermostable and glucose-tolerant *β*-glucosidase from *Fervidobacterium islandicum*. *Applied Microbiology and Biotechnology*.

[14] Pei J., Pang Q., Zhao L., Fan S., Shi H. (2012). Thermoanaerobacterium thermosaccharolyticum *β*-glucosidase: a glucose-tolerant enzyme with high specific activity for cellobiose. *Biotechnology for Biofuels*.

[15] Park A.-R., Kim H.-J., Lee J.-K., Oh D.-K. (2010). Hydrolysis and transglycosylation activity of a thermostable recombinant *β*-glycosidase from *Sulfolobus acidocaldarius*. *Applied Biochemistry and Biotechnology*.

[16] Kim H.-J., Park A.-R., Lee J.-K., Oh D.-K. (2009). Characterization of an acid-labile, thermostable *β*-glycosidase from *Thermoplasma acidophilum*. *Biotechnology Letters*.

[17] Prokofeva M. I., Kostrikina N. A., Kolganova T. V. (2009). Isolation of the anaerobic thermoacidophilic crenarchaeote *Acidilobus saccharovorans* sp. nov. and proposal of *Acidilobales* ord. nov., including *Acidilobaceae* fam. nov. and *Caldisphaeraceae* fam. nov. *International Journal of Systematic and Evolutionary Microbiology*.

[18] Mardanov A. V., Svetlitchnyi V. A., Beletsky A. V. (2010). The genome sequence of the crenarchaeon *Acidilobus saccharovorans* supports a new order, *Acidilobales*, and suggests an important ecological role in terrestrial acidic hot springs. *Applied and Environmental Microbiology*.

[19] Craven G. R., Steers E., Enfinsen C. B. (1965). Purification, composition and molecular weight of the *β*-galactosidase of *Escherichia coli* K12. *The Journal of Biological Chemistry*.

[20] Bowden A. C. (1974). A simple graphical method for determining the inhibition constants of mixed, uncompetitive and non competitive inhibitors. *Biochemical Journal*.

[21] Pisani F. M., Rella R., Raia C. A. (1990). Thermostable *β*-galactosidase from the archaebacterium *Sulfolobus solfataricus*. Purification and properties. *European Journal of Biochemistry*.

[22] Kengen S. W. M., Luesink E. J., Stams A. J. M., Zehnder A. J. B. (1993). Purification and characterization of an extremely thermostable *β*-glucosidase from the hyperthermophilic archaeon *Pyrococcus furiosus*. *European Journal of Biochemistry*.

[23] Ezaki S., Miyaoku K., Nishi K.-I. (1999). Gene analysis and enzymatic properties of thermostable *β*-glycosidase from *Pyrococcus kodakaraensis* KOD1. *Journal of Bioscience and Bioengineering*.

[24] Haq I. U., Khan M. A., Muneer B. (2012). Cloning, characterization and molecular docking of a highly thermostable *β*-1,4-glucosidase from *Thermotoga petrophila*. *Biotechnology Letters*.

[25] Bhatia Y., Mishra S., Bisaria V. S. (2002). Microbial *β*-glucosidases: cloning, properties, and applications. *Critical Reviews in Biotechnology*.

[26] Grogan D. W. (1991). Evidence that *β*-galactosidase of *Sulfolobus solfataricus* is only one of several activities of a thermostable *β*-D-glycosidase. *Applied and Environmental Microbiology*.

[27] Trofimov A. A., Polyakov K. M., Tikhonov A. V. (2013). Structures of *β*-glycosidase from *Acidilobus saccharovorans* in complexes with tris and glycerol. *Doklady Biochemistry and Biophysics*.

[28] Biely P. (1985). Microbial xylanolytic systems. *Trends in Biotechnology*.

[29] Moreira L. R. S., Filho E. X. F. (2008). An overview of mannan structure and mannan-degrading enzyme systems. *Applied Microbiology and Biotechnology*.

[30] Viikari L., Alapuranen M., Puranen T., Vehmaanperä J., Siika-Aho M. (2007). Thermostable enzymes in lignocellulose hydrolysis. *Advances in Biochemical Engineering/Biotechnology*.

